# Artificial intelligence applied to analyzes during the pandemic: COVID-19 beds occupancy in the state of Rio Grande do Norte, Brazil

**DOI:** 10.3389/frai.2023.1290022

**Published:** 2023-12-08

**Authors:** Tiago de Oliveira Barreto, Nícolas Vinícius Rodrigues Veras, Pablo Holanda Cardoso, Felipe Ricardo dos Santos Fernandes, Luiz Paulo de Souza Medeiros, Maria Valéria Bezerra, Filomena Marques Queiroz de Andrade, Chander de Oliveira Pinheiro, Ignacio Sánchez-Gendriz, Gleyson José Pinheiro Caldeira Silva, Leandro Farias Rodrigues, Antonio Higor Freire de Morais, João Paulo Queiroz dos Santos, Jailton Carlos Paiva, Ion Garcia Mascarenhas de Andrade, Ricardo Alexsandro de Medeiros Valentim

**Affiliations:** ^1^Laboratory of Technological Innovation in Health (LAIS), Federal University of Rio Grande do Norte (UFRN), Natal, Rio Grande do Norte, Brazil; ^2^Advanced Nucleus of Technological Innovation (NAVI), Federal Institute of Rio Grande do Norte (IFRN), Natal, Rio Grande do Norte, Brazil; ^3^Federal Institute of Rio Grande do Norte (IFRN), Ceará-Mirim, Rio Grande do Norte, Brazil; ^4^Secretary of Public Health of Rio Grande do Norte, Natal, Rio Grande do Norte, Brazil; ^5^Brazilian Company of Hospital Services (EBSERH), University Hospital of Pelotas, Federal University of Pelotas (UFPel), Pelotas, Rio Grande do Sul, Brazil

**Keywords:** machine learning, deep learning, computational methods, bed regulation, COVID-19, RegulaRN

## Abstract

The COVID-19 pandemic is already considered one of the biggest global health crises. In Rio Grande do Norte, a Brazilian state, the RegulaRN platform was the health information system used to regulate beds for patients with COVID-19. This article explored machine learning and deep learning techniques with RegulaRN data in order to identify the best models and parameters to predict the outcome of a hospitalized patient. A total of 25,366 bed regulations for COVID-19 patients were analyzed. The data analyzed comes from the RegulaRN Platform database from April 2020 to August 2022. From these data, the nine most pertinent characteristics were selected from the twenty available, and blank or inconclusive data were excluded. This was followed by the following steps: data pre-processing, database balancing, training, and test. The results showed better performance in terms of accuracy (84.01%), precision (79.57%), and F1-score (81.00%) for the Multilayer Perceptron model with Stochastic Gradient Descent optimizer. The best results for recall (84.67%), specificity (84.67%), and ROC-AUC (91.6%) were achieved by Root Mean Squared Propagation. This study compared different computational methods of machine and deep learning whose objective was to classify bed regulation data for patients with COVID-19 from the RegulaRN Platform. The results have made it possible to identify the best model to help health professionals during the process of regulating beds for patients with COVID-19. The scientific findings of this article demonstrate that the computational methods used applied through a digital health solution, can assist in the decision-making of medical regulators and government institutions in situations of public health crisis.

## 1 Introduction

The COVID-19 pandemic is already considered one of the biggest global health crises of the century (Hu et al., 2021). The first cases appeared in the city of Wuhan, China, and in December 2019, the disease quickly spread among the other continents of the world, manifesting itself as a dry cough, fever, and fatigue (Huang et al., 2020; Zhu et al., 2020; Dashboard, 2022). In Brazil, the first confirmed cases appeared in February 2020 and, the following month it was declared a pandemic situation (Aquino et al., 2020; Bastos and Cajueiro, 2020; Costa et al., 2021; Valentim et al., 2021).

Statistical data indicate that about 82% of COVID-19 patients have the mildest symptoms of the disease. However, the evolution to the most serious phase causes severe acute respiratory syndrome, pneumonia, and multiple organ failure, requiring hospitalization inward or intensive care unit (ICU) beds (Ahsan et al., 2020; Sales-Moioli et al., 2022). In Brazil, more than 60 thousand ICU beds were made available to treat patients, however, there were still more than 680 thousand deaths (Cotrim Junior and Cabral, 2020; Dashboard, 2022). Thus, with the daily increase in case numbers, several officials had difficulties in monitoring and providing access to beds for patients (Perondi et al., 2021; Shahzad et al., 2022).

In addition, the Brazilian Northeast region faced another aggravating factor, as it is historically one of the regions with the lowest healthcare resources, and consequently presented the second lowest proportion of bed availability per capita (Cotrim Junior and Cabral, 2020; Lino et al., 2020). According to Valentim et al. (2021), expectations of coping with COVID-19 for a state like Rio Grande do Norte (RN) in the Northeast Region of Brazil, were quite pessimistic. One of the support measures taken by the federal government, specifically the Ministry of Health (MoH), was the provision of financial resources to reduce the effects of the pandemic. However, some states received less funding regionally, with RN being one of the states that obtained the lowest amount of resources per capita, as available at the transparency portal of the federal government of Brazil itself (https://www.portaltransparencia.gov.br/coronavirus?ano=2020).

In this context, considering the difficulty of monitoring and regulating access to beds for COVID-19 patients, particularly due to high pressure (demand for beds), the State Secretariat of Public Health of Rio Grande do Norte (SESAP/RN) in Brazil, through technical-scientific cooperation with the Laboratory for Technological Innovation in Health (LAIS) developed a digital health solution to mitigate these problems. This solution was called RegulaRN Platform, whose objective was to monitor and control access to clinical and intensive care unit (ICU) beds for patients with COVID-19 in the State of RN/Brazil (Valentim et al., 2021; Sales-Moioli et al., 2022). Furthermore, the RegulaRN Platform acted as a tool for public transparency by publishing online several indicators on COVID-19, daily data used by the local press and the national press consortium to disseminate the epidemiological scenarios of the State (Valentim et al., 2021).

The bed regulation process is a critical sector in public health management, as it acts directly in the management of sectors that can impact the lives of patients, especially those who need hospitalization in emergency cases (Maldonado et al., 2021). Therefore, it is necessary to develop techniques that can contribute to the continuous improvement of the work process of health professionals working at this healthcare level.

In response to the challenges pointed out, the use of digital health solutions based on intelligent computational methods can help reduce impacts and enhance better decision-making by public agencies, especially in situations of a public health crisis, such as the COVID-19 pandemic (Shailaja et al., 2018; Bian and Modave, 2020). Intelligent computational models, when well applied and aligned with a good governance policy, can contribute in a more effective way, to promote the reduction of uncertainties, ambiguities, subjective gaps, and better support for decision-making (Ghaderzadeh and Aria, 2021; Moulaei et al., 2022). Moreover, these models are able to identify non-linear relationships and interactions between variables, which enables better performance of the systems where they are applied (Subudhi et al., 2020).

Thus, the research presented in this article aimed, firstly, to analyze a set of data from a bed regulation system, used during a period of the COVID-19 pandemic. Secondly, select and compare different machine learning and deep learning models, to elect the most relevant classification model, which can predict with the best accuracy, precision, recall, specificity, F1-score, and ROC-AUC curve, the mortality of patients with COVID-19 who were regulated to be hospitalized. Finally, to discuss the potential of this tool in the decision-making of health professionals and public entities during a public health crisis situation, such as the one seen during the COVID-19 pandemic.

## 2 Materials and methods

The methodological procedure used in this study consisted of analyzing the regulation data profile for data extraction, features selection, classification of data, and cleaning data. Then the data were analyzed and use of the data for application in computational models which involved five steps: (1) definition of evaluation metrics, (2) data balance, (3) segmentation of training and validation data, (4) definition of data classification models, and (5) definition of hyperparameters for training. In the following topics, the data will be presented and discussed from the perspective of using computational intelligence tools to support decision-making in the health axis.

### 2.1 Data extraction and preprocessing

This study used the RegulaRN COVID-19 bed regulation database, a system used to meet the regulatory flows of Rio Grande do Norte (RN)/Brazil. The database was extracted on 08/02/2022, when it contained 25,366 effective regulations, considering the two health regions of the State: West and Metropolitan. The data sample covered the period from April 30, 2020, to August 2, 2022.

The raw database (before preprocessing) had twenty characteristics, of which nine were considered relevant for data classification because they were data more correlated to the patient's clinical condition. Table 1 presents all the data present in the database and their respective descriptions. Characteristics that referred to: (a) request date, (b) patient's municipality, (c) patient's federal unit, (d) if pregnancy, (e) gestational age, (f) date of entry to the bed, (g) date of output from the bed, (h) requesting hospital unit, (i) municipality of the requesting hospital unit, (j) providing hospital unit, and (k) municipality of the providing hospital, because they are not definitive for determining the outcome and this could affect the results of the study. In this way, the characteristics that were most related to the patient's health condition and the bed offered were considered, as follows: (a) age, (b) case type, (c) Unified Prioritization Score (EUP score), (d) if on orotracheal intubation (OTI), (e) type of bed requested, (f) type of entrance bed, (g) type of output bed, (h) length of stay, and (i) the outcome; because they are variables that have more correspondence with the outcome (target).

**Table 1:** Description of database.

**Data description**	
**Field**	**Description**
Request date	Represents the date a bed request was registered.
Patient's municipality	Represents the patient's municipality.
Patient's federal unit	Represents the patient's federal unit.
Pregnant	Represents whether the patient is pregnant.
Gestational age	Describe how far along the pregnancy is, measured in weeks.
Age	Represents the patient's age.
Case type	Represents whether the patient was “suspected,” “confirmed,” or “discarded” for COVID-19.
EUP score	Represents the EUP score value.
OTI	Represents whether or not the patient was in orotracheal intubation when requesting a bed.
Requested bed type	Represents the type of bed that was selected by the regulation center for a patient.
Entrance date	Represents the date that the patient was allocated in the health unit (hospital).
Entrance bed type	Represents the type of bed that the patient was allocated in the health unit (hospital).
Output date	Represents the date that the patient left the bed after the outcome.
Output bed type	Represents the type of bed the patient was in before the outcome.
Length of stay	Hospital length of stay, measured in days.
Outcome	Represents the final outcome of the patient in bed.
Requesting unit	Represents the unit health that solicits a bed for the patient.
Municipality of the requesting unit	Represents the municipality of the health unit that solicits the bed.
Provider unit	Represents the unit health that receives and internal the patient in the bed.
Municipality of the provider unit	Represents the municipality of the health unit that receives and internal the patient in the bed.

**Figure 1:**
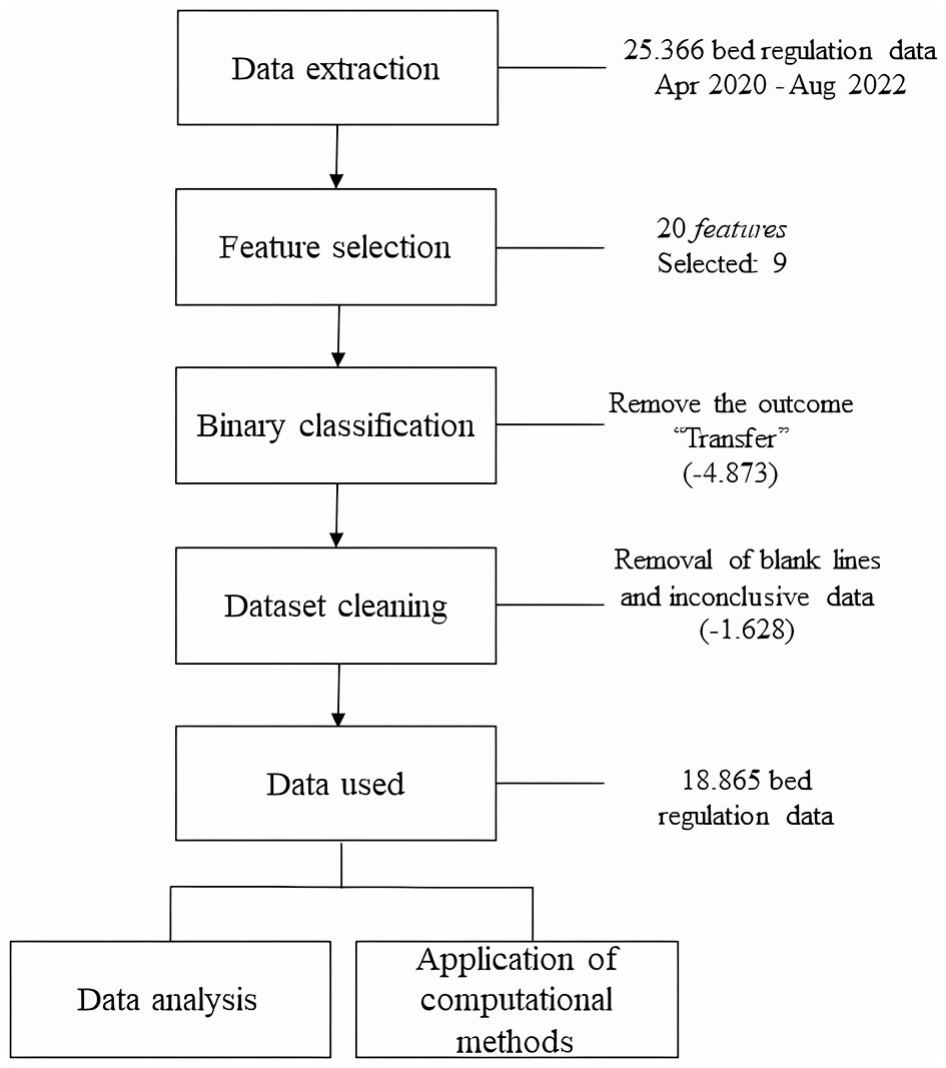
Pipeline for the use of RegulaRN data.

The determination of the nine pivotal characteristics, delineated as Feature Selection in Figure 1, was guided by insightful consultations with clinical specialists, thereby categorizing our feature selection approach as specialist-driven. It is imperative to note that feature selection strategies are encompassed within the overarching strategy known as dimensionality reduction, which concurrently includes a subset of methodologies recognized in academic literature as feature extraction (Jia et al., 2022). Our methodology hinged on the exclusive employment of a feature selection strategy, substantiated by its intrinsic ability to preserve the clinical interpretability of the variables, thereby enhancing the model's explicability—a facet of paramount importance in medical and public health applications. Although feature extraction techniques like Principal Component Analysis (PCA) are renowned for their adeptness in dimensionality reduction, they also inherently possess a disadvantage, notably, the potential for the newly derived features to lose their original clinical meaning (Jia et al., 2022). This facet is not favorable for our application in the clinical domain, where preserving the comprehensibility and interpretability of variables is crucial.

In terms of preparing the dataset, to normalize and standardize the data, lines in the database with blank data or with incorrectly registered information were removed. In addition, the “outcome” characteristic could assume three results: “discharge,” “death,” and “transfer.” However, the outcome “transfer” indicates that the patient was transferred from one hospital to another or from one bed to another. Therefore, it is not a determining factor as to whether the patient was discharged or died at the end of hospitalization. Thus, lines with “Transfer” as the outcome were removed, and the final definition was kept as a binary classification (“discharge” or “death”). In the end, 74.37% of the data was kept, representing 18,865 effective regulations (inpatients). To ensure the reproducibility of the experiment, the database with all the pre-processed information is available in the following repository https://zenodo.org/record/8122564. According to Resolution 674, 2022 of the National Health Council (NHC) of the Ministry of Health (MoH) this research is exempt from registration with the Research Ethics Committee (CEP)/Brazil or the National Research Ethics Commission (CONEP)/Brazil, as it works with databases, whose information is gathered without the possibility of individual identification.

Once the dataset elements had been defined, analyses were carried out to evaluate and characterize them for use in the models of the computational methods to be selected. Figure 1 shows schematically how the procedures described were carried out.

### 2.2 Analysis and correlation between dataset features

The data analyzed was the same as that selected for the computer model applications so that it was divided into categories and outcomes. During the analysis, a statistical evaluation was carried out with a 99% confidence interval (*p*-value < 0.01) between the selected characteristics and also segmented by the outcome.

A possible relation was found between two independent variables, age, and length of stay, which do not have direct causality with the outcome. For these two data, the chi-square statistical distribution was applied (CI 99%, (*p* < 0.01) and from this, we can categorize the different risk criteria that are linked to the outcome.

To assess the correlation between the variables we used the Phik correlation (https://phik.readthedocs.io/). The Phik correlation is a variation of the Pearson hypothesis test with some refinements, such as the correlational evaluation of categorical, ordinal, and interval features. As 67% of the features in the dataset use categorical (non-numerical) values, the Phik correlation was selected for the correlational presentation of the data. We used the *pandas-profilereports* library (https://github.com/ydataai/ydata-profiling) to identify direct proportional correlations between the selected variables. The correlation matrix obtained was compared with the feature importances of the computational models to analyze a relational convergence between the data used.

### 2.3 Evaluation metrics

**Figure 2:**
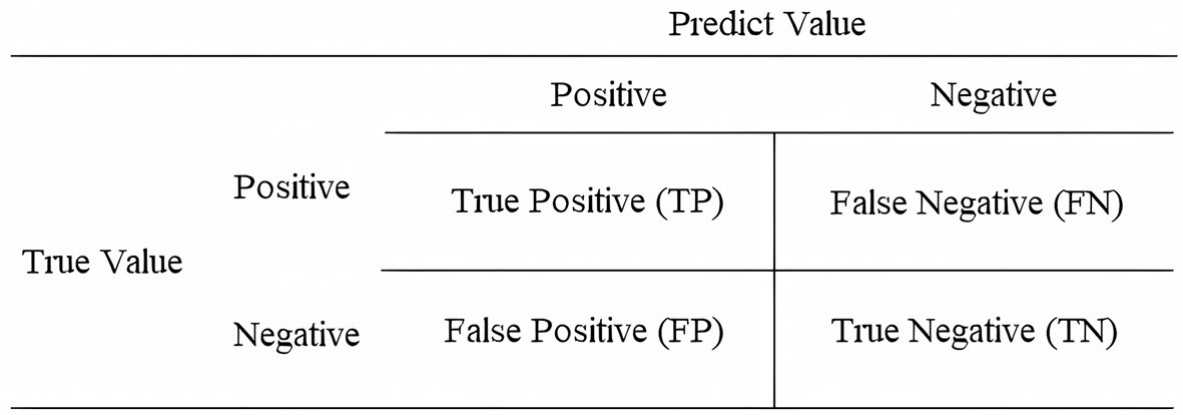
Structure of the confusion matrix.

The evaluation metrics used in this study were similar to other research, which used machine learning and/or deep learning, presented in the works of Sokolova and Lapalme (2009), Ghaderzadeh et al. (2021b), and Endo et al. (2022), namely: accuracy, precision, recall, F1-score, specificity, and ROC-AUC curve. These metrics are formulated from the confusion matrix. The confusion matrix shows the relationship between the real event and the prediction suggested by the model (Sokolova and Lapalme, 2009; Grandini et al., 2020). The composition of the confusion matrix consists of true positive (TP)—when the event is positive and the model predicts positive; false positive (FP)—when the event is negative and the model predicts positive; false negative (FN)—when the event is positive and the model predicts negative; and true negative (TN)—when the event is negative and the model predicts negative. Figure 2 illustrates the confusion matrix.

In this respect, the accuracy of a model relates to the number of data points that were predicted correctly, compared to the total number of possibilities. Thus, it is calculated from the sum of TP and TN, divided by the sum of possible events (Equation 1).


(1) $$\begin{array}{l} Accuracy=\left(TP+TN\right)/\left(TP+FP+FN+TN\right) \end{array}$$

Precision involves predicting the number of truly positive classification data compared to what the model has judged to be positive. It is calculated by dividing TP by the sum of TP and FP (Equation 2).


(2) $$\begin{array}{l} Precision=TP/\left(TP+FP\right) \end{array}$$

Recall consists of predicting the number of truly positive rating data compared to what the model has judged to be positive and negative. It is calculated by dividing TP by the sum of TP and FN (Equation 3).


(3) $$\begin{array}{l} Recall=TP/\left(TP+FN\right) \end{array}$$

Specificity refers to the correct prediction of truly negative values from the database. It is calculated by dividing TN by the sum of TN and FP (Equation 4).


(4) $$\begin{array}{l} Specificity=TN/\left(TN+FP\right) \end{array}$$

The F1-score is the harmonic mean of precision and recall. For learning systems with greater vulnerability, to further optimize the Recall and Precision values, the F1-score can be used (Lipton et al., 2014; Endo et al., 2022). The formula involves the product of precision and recall divided by the sum of these metrics, multiplied by 2 (Equation 5).


(5) $$\begin{array}{l} F1Score=2*\left(Precision*Recall\right)/\left(Precision+Recall\right) \end{array}$$

The ROC (Receiver Operating Characteristic) is obtained from the ratio between the true positive rate (TP/TP+FN) and the false positive rate (FP/FP+TN). Overall, it can be obtained by dividing the recall value by the complement of the specificity (Equation 6).


(6) $$\begin{array}{l} ROCAUC=Recall/\left(1-Specificity\right) \end{array}$$

### 2.4 Data balancing

One of the main problems when working with machine learning is data imbalance. This phenomenon occurs when there is a disproportionate categorization of data (Moulaei et al., 2022). RegulaRN's data composition is naturally unbalanced due to the low lethality of COVID-19. Therefore, in the hospitalization process, an asymmetry between the number of discharges and deaths is to be expected. As for the data used, 72% of the outcomes were classified as discharges and 28% as deaths. It should be noted that all the data used in this experiment is from the real world, i.e., from real patients who have been infected with the SARS CoV-2 virus, who have developed the moderate or more severe form of the COVID-19 disease and have needed to be hospitalized for more appropriate care. Thus, it is not patient data that has been previously selected for training the algorithms, the database used was built organically, from bed regulations for COVID-19 patients.

An imbalanced base may therefore have higher hits for metrics in which the most representative class is more dominant, which can be a negative aspect, as the algorithms or computer models may act in a discriminatory way. To reduce instability, the SMOTE (Synthetic Minority Over-sampling) balancing method was used. The SMOTE consists of increasing the sample of the minority class and minimizing the majority class, producing an over-sample (Chawla et al., 2002; Fernández et al., 2018). Thus, the SMOTE makes the sample used in the experiment less asymmetrical concerning the classes.

### 2.5 Training and test data

The separation process of training and validation data in this study corroborates the methodological procedures found in other similar studies with a significant volume of data. The study by Endo et al. (2022) used around 11,382 data points with classifier models to make predictions about the dissemination of information about COVID-19. Meanwhile, Vaughan et al. (2023) used more than 10,849 wastewater samples from different European regions to predict COVID-19 contamination. Yu et al. (2021) used 5,471 datas of patients with COVID-19 to predict mortality and mechanical ventilation.

Thus, considering the large volume of data processed and the scientific approach of separating the data in the model into 80% for training and 20% for test, the same method was used in this scientific study.

### 2.6 Definition of models for data classification

At first, the process of selecting classification models involved algorithms that according to the literature performed well with a high volume of data (Charbuty and Abdulazeez, 2021; Yu et al., 2021). Thus, two classical models of machine learning were selected, the decision tree and random forest algorithms, and the deep learning model with the multilayer perceptron (MLP) algorithm. In MLP, to obtain better computational performance, especially since there is a large volume of data times the number of variables (this increases the dimensions in the hyperplane), it is necessary to select optimizing algorithms during the training stage (Sutskever et al., 2013; Yu et al., 2021). Optimizing algorithms are used to improve time and accuracy in the data classification process. Therefore, the following optimization algorithms were selected: Stochastic Gradient Descent (SGD) (Zhang et al., 2018), Adam (Kingma and Ba, 2014), Adagrad (Duchi et al., 2011), and Root Mean Square Propagation (RMSprop) (Dauphin et al., 2015).

The selected models were applied to a training pipeline with the aim of selecting the best hyperparameters and maximizing the selected metrics, as well as evaluating the importance of the variables for the model's decision.

### 2.7 Hyperparameters for defining the best model

In machine and deep learning, hyperparameters are variables that help in the definition of classifiers during learning, they cannot be altered during training (Yu et al., 2021). Table 2 shows the hyperparameters used in each model and the range of values adopted for the experiment. The highlighted values show the best-performing combinations.

**Table 2:** Hyperparameter segmentation by model.

**Models**	**Hyperarameter**	**Levels**
**Machine learning**		
Decision tree	Criterion	Gini or **entropy**
	Max depth of the tree	[**10**, 50, 100]
	Min samples leaf	Range [1,2,**3**,4]
	Max features	[**sqrt**, log2]
Random forest	Criterion	Gini or **entropy**
	Max depth of the tree	[**10**, 50, 100]
	Number estimators	[**100**, 200, 400]
	Max features	[**sqrt**, log2]
**Deep learning**		
SGD	Number of neurons	[5, 20, **100**]
	Dropout	[**0.5**, 0.9]
	Batch size	[**16**, 32, 64]
	Epochs	[10, **50**, 100]
Adam	Number of neurons	[5, 20, **100**]
	Dropout	[**0.5**, 0.9]
	Batch size	[**16**, 32, 64]
	Epochs	[10, 50, **100**]
RMSprop	Number of neurons	[5, 20, **100**]
	Dropout	[**0.5**, 0.9]
	Batch size	[**16**, 32, 64]
	Epochs	[10, **50**, 100]
Adagrad	Number of neurons	[5, 20, **100**]
	Dropout	[**0.5**, 0.9]
	Batch size	[**16**, 32, 64]
	Epochs	[10, 50, **100**]

In bold, the hyperparameter values that generated better average values for the metrics are highlighted.

For the Decision Tree model—executed by DecisionTreeClassifier from the sckitlearn python library Pedregosa et al. (2011), the hyperparameters used were: *criterion, max depth of the tree, min samples leaf*, and *max features*. As for the *criterion*, the possibilities adopted were gini or entropy, which are mathematical tools that calculate the possibility of incorrect classification of a given characteristic. Depending on the homogeneity of the data, there may be variations in the final results. The *max depth of the tree* considers the number of nodes from the root to the furthest element. The *min samples leaf* considers the smallest possible number of samples for a node. The *max features* take into account the number of features that must be used in each operation. In the Random Forest model, sckitlearn's RandomForestClassifier library was used to select the hyperparameters: *criterion, max depth of the tree, number estimators*, and *max features*. The *number estimator* parameter considers the number of forest trees.

For the models using the multilayer perceptron, the python Keras library (https://keras.io/) was applied, and the hyperparameters selected: *number of neurons, dropout, batch size*, and *epochs*. The *number of neurons* represents the number of neurons in each hidden layer of the perceptron. *Dropout* acts to select the number of neurons that will indeed be active in a hidden layer. *Batch size* involves the number of examples used to estimate the error gradient before updating the model parameters. *Epochs* means the number of complete passes through the data set before the training process is terminated.

For all the models, the GridSearchCV tool, a Python library, was used to explore all possible possibilities to find the best parameters for the grid (Ensor and Glynn, 1997; Bergstra and Bengio, 2012). In addition, the cross-validation method was used with a value of 10 (Moulaei et al., 2022), and the models were run five times, a method used in other academic works, such as Ahsan et al. (2020), to guarantee the selection of the best parameters even if there was variation in any of them. For each run, we calculate the average values and standard deviation of the metrics during the test stage.

## 3 Results

### 3.1 Results of data pre-processing and characterization

As for the data profile, Table 3 shows the number of patients classified by their outcome (divided into percentage and absolute values) and divided by the main characteristics selected. According to this exploration, it is possible to identify that the number of deaths is proportionally higher in patients aged over 60, who had a higher EUP score, who started the hospitalization process already intubated, who used an ICU bed and required a hospital stay of more than 7 days.

**Table 3:** Hyperparameter segmentation by model.

**Features**		**Values**	**Outcome**
Age	(≥) 60	9,116 (48.3%)	Discharge: 5,599 (61,4%) Death: 3,517 (38,6%)
	(< ) 60	9,749 (51.7%)	Discharge: 8,016 (82.2%) Death: 1,733 (17.8%)
Type of case	Confirmed	13,235 (70.2%)	Discharge: 9,158 (69.1%), Death: 4,077 (30.9%)
	Suspect	5,150 (27.3%)	Discharge: 4,100 (79.6%) Death: 1,050 (20.4%)
	Discarded	480 (2.5%)	Discharge: 357 (74.4%) Death: 123 (25.6%)
EUP score	2	9,100 (48.2%)	Discharge: 7,259 (79.8%), Death: 1,839 (20.2%)
	3	4,138 (21.9%)	Discharge: 3,088 (74.6%) Death: 1,050 (25.4%)
	4	2,554 (13.5%)	Discharge: 1,698 (66.5%) Death: 856 (33.5%)
	5	2,056 (10.8%)	Discharge: 1,118 (54.4%) Death: 938 (45.6%)
	6	781 (4.1%)	Discharge: 359 (46%) Death: 422 (54%)
	7	190 (1%)	Discharge: 72 (37.9%) Death: 118 (62.1%)
	8	46 (0.2%)	Discharge: 20 (43.5%) Death: 26 (56.5%)
IOT	No	17,634 (93.4%)	Discharge: 13,434 (76.2%), Death: 4,202 (23.8%)
	Yes	1,231 (6.5%)	Discharge: 183 (14.9%) Death: 1,048 (85.1%)
Requested bed	Ward	11,289 (59.8%)	Discharge: 10,064 (89.1%), Death: 1,225 (10.9%)
	ICU	7,576 (40.2%)	Discharge: 3,551 (46.9%) Death: 4,025 (53.1%)
Entrance bed	Ward	9,753 (51.7%)	Discharge: 8,800 (90.2%), Death: 953 (9.8%)
	ICU	9,112 (48.3%)	Discharge: 4,815 (52.8%) Death: 4,297 (47.2%)
Output bed	Ward	10,511 (55.7%)	Discharge: 10,077 (95.9%), Death: 434 (4.1%)
	ICU	8,354 (44.3%)	Discharge: 3,538 (42.4%)Death: 4,816 (57.6%)
Length of stay	(< ) 7	11,852 (62.8%)	Discharge: 9,293 (78.4%), Death: 2,559 (21.6%)
	7 (≤) LoS (≤) 14	5,038 (26.7%)	Discharge: 3,365 (66.8%) Death: 1,673 (33.2%)
	(>) 14	1,975 (10.5%)	Discharge: 957 (48.5%) Death: 1,018 (51.5%)
Outcome	Discharge	13,615 (72.1%)	
	Death	5,250 (27.9%)	

**Figure 3:**
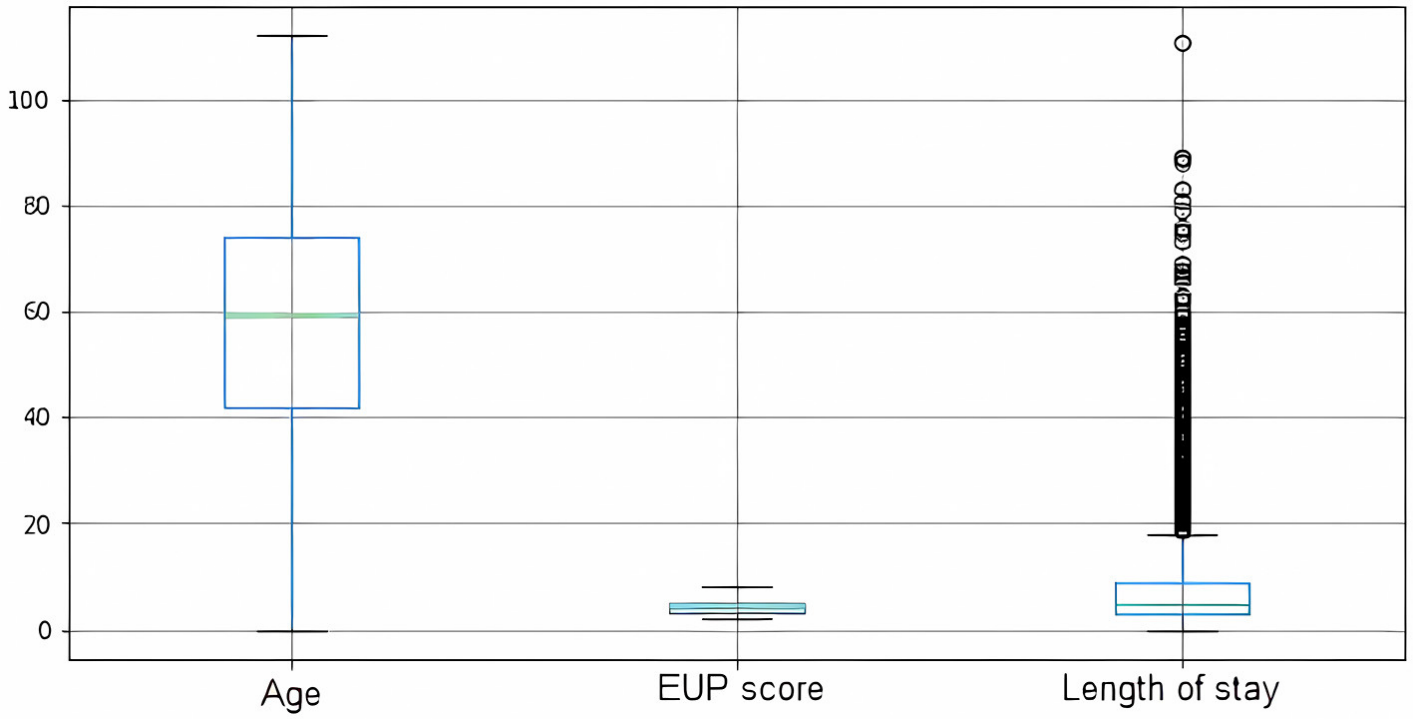
Boxplot representation of numerical data.

With regard to the statistical profile, when evaluating the data as a whole, the average age of the patients who needed some kind of hospitalization was 55.8 years, with a standard deviation of 23.7. The median age was 59, which indicates that the average population of RN needing hospitalization is an intermediate age group close to the elderly. As for the EUP score, the mean value was 3 and the median also had the same value. It can therefore be said that patients who were regulated on the platform had lower EUP scores and therefore fewer health complications. Concerning the length of stay, the average length of stay was 6.87 days, with a standard deviation of 7 days. Figure 3 shows a distribution of these data in a boxplot.

The literature does not provide an accurate definition of the expected length of stay for a patient with COVID who will need hospitalization, because this determination depends on several factors such as the patient's current state of health, the assistance capacity of the place of care, and socio-regional factors. However, academic studies indicate a length of stay of between 5 and 29 days (Rees et al., 2020; Vekaria et al., 2021). The RegulaRN data shows some outliers in the length of stay (Figure 3) that can be evaluated in future studies on the efficiency of care utilization.

**Figure 4:**
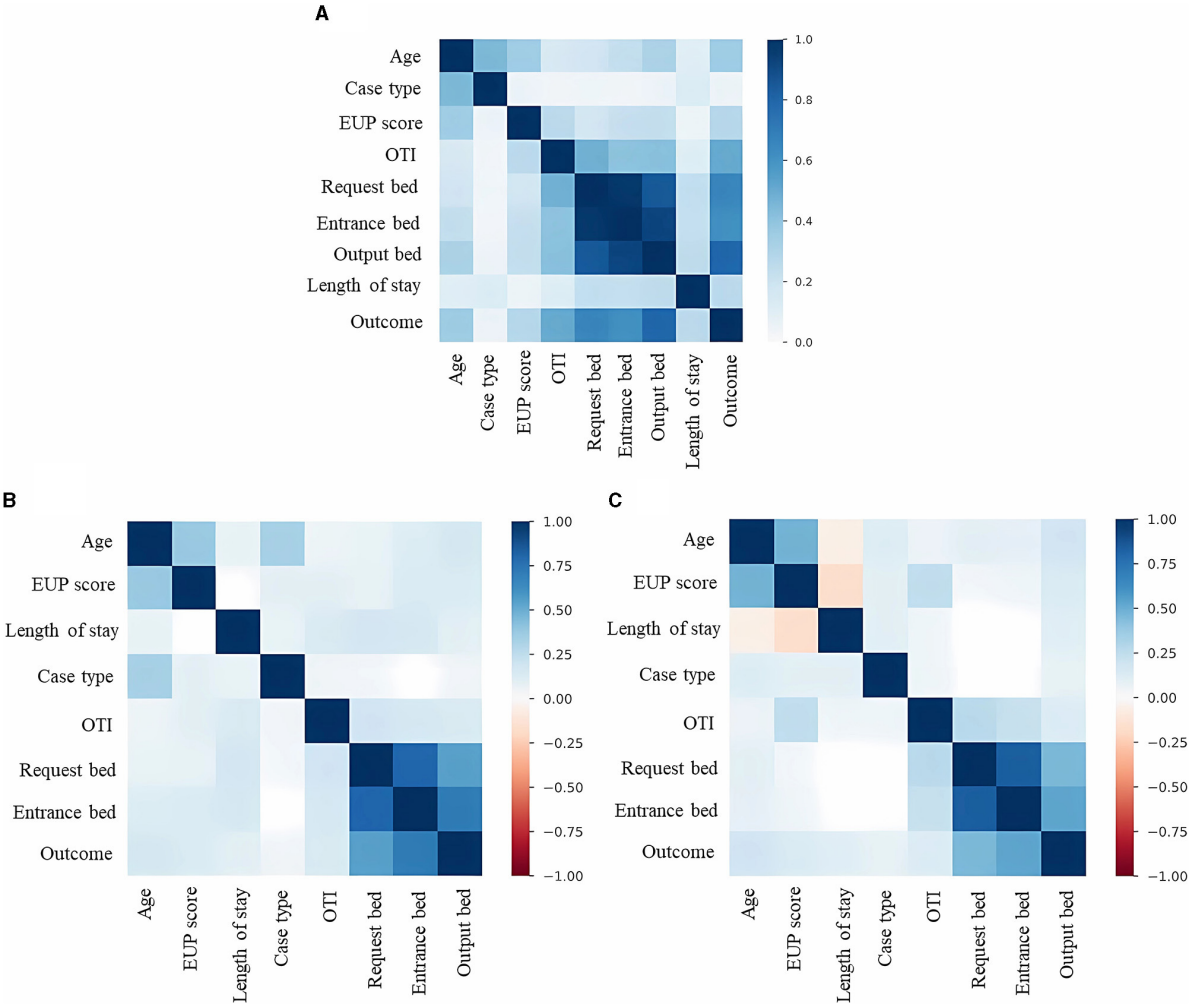
Correlation between dataset features. **(A)** Correlations between the characteristics of the dataset involving discharge and death results. **(B)** Correlations between the characteristics of the dataset involving only the discharge results. **(C)** Correlations between the characteristics of the dataset involving only the death results.

As for the level of correlation between the variables (Figure 4), evaluating the entire database (Figure 4A), the pandas-profilereports function manages to identify directly proportional correlations between the selected variables, so that age, EUP score, orotracheal intubation, requested bed, entrance bed, and output bed appear to be more relevant in defining the outcome. When evaluating the dataset based on the outcome, it can be seen that the behavior of the data when the final outcome is “discharge” (Figure 4B) is similar to the complete data structure. This result is to be expected given that the predominant volume of data is from patients who had “discharge” outcomes. However, when evaluating the structure of data whose outcome was “death” (Figure 4C), it is possible to identify the existence of negative correlations between the variables age, length of stay, and EUP score. This information also points to expected narratives, given that older patients tend to spend less time in the hospital (when correlating age and length of stay), just as patients with more fragile health, who score higher on the EUP score, are also more likely to die in a shorter period.

Evaluating the data profile by discharge and death (Table 4), non-survivors are older, have higher scores on the EUP score, started hospitalization already under orotracheal intubation have a higher indication for an ICU bed, and stay for more than eight days in hospital on average. Survivors are younger, have lower scores on the EUP score, started hospitalization without orotracheal intubation, have a higher indication for a ward bed, and stay less than 7 days in hospital.

**Table 4:** Patient data profile divided by discharge and death.

	**Discharge**	**Death**	**99% CI (*****p*****-value**<**0,01)**	
			**Discharge**	**Death**
**Total**	13,615 (72.3%)	5,250 (27.8%)	-	-
**Age**	51.69 (±24.43)	66.47 (±17.86)	[51.142, 52.238]	[65.835, 67.105]
**Case type**				
Confirmed	9,158 (69.1%)	4077 (30.9%)	[0.679, 0.703]	[0.290, 0.328]
Suspect	4,100 (79.6%)	1050 (20.4%)	[0.779, 0.812]	[0.172, 0.236]
Discarded	357 (74.4%)	123 (25.6%)	[0.685, 0.803]	[0.155, 0.357]
**EUP score**				
2	7,259 (79.8%)	1,839 (20.2%)	[0.786, 0.810]	[0.178, 0.226]
3	3,088 (74.6%)	1,050 (25.4%)	[0.726, 0.766]	[0.219, 0.289]
4	1,698 (66.5%)	856 (33.5%)	[0.636, 0.694]	[0.294, 0.376]
5	1,118 (54.4%)	938 (45.6%)	[0.506, 0.582]	[0.414, 0.498]
6	359 (46%)	422 (54%)	[0.392, 0.528]	[0.478, 0.602]
7	72 (37.9%)	118 (62.1%)	[0.232, 0.526]	[0.506, 0.736]
8	20 (43.5%)	26 (56.5%)	[0.150, 0.720]	[0.315, 0.815]
**OTI**				
Yes	183 (14.9%)	1,048 (85.1%)	[0.081, 0.217]	[0.823, 0.880]
No	13,434 (76.2%)	4,202(23.8%)	[0.752, 0.771]	[0.221, 0.255]
**Requested bed**				
Ward	10.064 (89.1%)	1,225 (10.9%)	[0.883, 0.899]	[0.086, 0.132]
UCI	3,551 (46.9%)	4,025 (53.1%)	[0.447, 0.491]	[0.511, 0.551]
**Entrance bed**				
Ward	8,800 (90.2%)	953 (9.8%)	[0.894, 0.910]	[0.073, 0.123]
UCI	4,815(52.8%)	4,297 (47.2%)	[0.510, 0.546]	[0.452, 0.492]
**Output bed**				
Ward	10,077 (95.9%)	434 (4.1%)	[0.954, 0.964]	[0.017, 0.065]
UCI	3,538 (42.4%)	4.816 (57.6%)	[0.403, 0.445]	[0.558, 0.594]
**Length of stay**	6,102 (±6,210)	8,789 (±8,583)	[5.966, 6.240]	[8.484, 9.094]

After applying the chi-square, we identified a strong correlation between the independent variables age and length of hospital stay. By making a selection based on the average length of hospitalization and age groups, it was able to establish risk criteria based on how these variables present themselves and thus help decision-making at the care level (Table 5). A patient who is over 80 years old and has been hospitalized for more than seven days has a 53% chance of death, reaching the maximum risk criterion. In this way, they can be relocated from an ICU bed to a palliative care bed.

**Table 5:** Categorization of risk criteria based on age and length of stay (LoS).

	**Discharge (%)**		**Death (%)**		**Risk criterion**
Age	LoS ≤ 7	LoS (>) 7	LoS ≤ 7	LoS (>)7	
≤ 49	89,64	73,54	10,35	26,45	1
50–59	81,44	61,74	18,55	38,25	2
60–69	74.59	54.04	25.40	45.95	3
70–79	68.65	51.56	31.34	48.43	4
≥80	58.53	46.68	41.47	53.31	5

### 3.2 Results of the application of computational methods

In Table 2, displayed in the materials and methods section, the selected hyperparameters and their respective defined values are shown. The best hyperparameters have been highlighted in bold so that it is possible to identify an equivalence between similar parameters in the different models. The machine learning models maintained better results with the same levels of *criterion, max depth of the tree*, and *max features*, this correspondence phenomenon also occurred among the deep learning models, in which there was the same selection of the *number of neurons, dropout*, and *batch size* among all the optimizers.

As for the evaluation metrics (Table 6), among the machine learning models, Random Forest showed the best accuracy (82.97%), precision (79.35%), recall (84.80%), F1-score (80.74%), and specificity (84.79%) with the lowest standard deviation values. In the deep learning models, the highest accuracy (84.01%), precision (79.57%), and F1-score (81%) were achieved by SGD, while RMSProp was responsible for the highest recall (84.67%) and specificity (84.67%). A significant variation in the standard deviation was found between the deep learning models.

**Table 6:** Categorization of risk criteria based on age and length of stay (LoS).

**Models**	**Accuracy**	**Precision**	**Recall**	**F1-score**	**Specificity**
**Machine learning**					
Decision tree	82.06(± 0.29)	78.41 (± 0.21)	83.72(± 0.26)	79.73 (± 0.26)	84.38 (± 0.85)
Random forest	**82.97(**±**0.11)**	**79.35 (**±**0.12)**	**84.80 (**±**0.16)**	**80.74 (**±**0.12)**	**84.79 (**±**0.19)**
**Deep learning**					
SGD	**84.01 (**±**0.25)**	**79.57 (**±**0.46)**	84.16 (+0.13)	**81.00 (**±**0.49)**	84.16 (+0.12)
Adam	82.84 (± 0.34)	79.20 (±0.20)	84.57(±0.22)	80.58(±0.24)	84.57(±0.23)
RMSprop	82.43(± 0.25)	78.97 (± 0.20)	**84.67 (**±**0.18)**	80.28 (±0.24)	**84.67 (**±**0.18)**
Adagrad	83.11 (±0.43)	79.38 (±0.29)	84.59 (±0.18)	80.80 (±0.35)	84.59(±0.18)

The highest values according to each metric are highlighted in bold.

**Figure 5:**
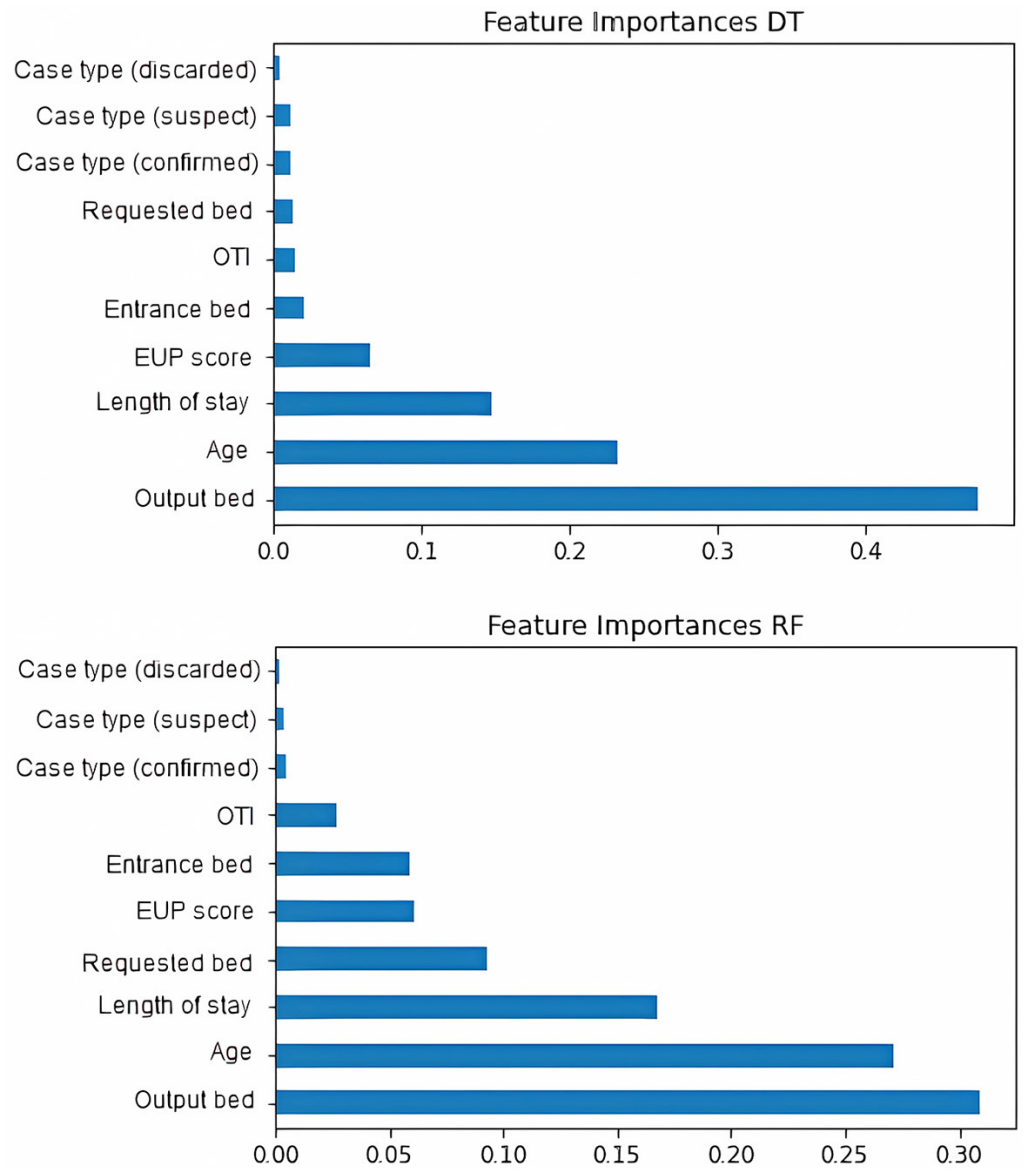
Features importances of machine learning models. The figure initially shows decision tree and then Random Forest.

**Figure 6:**
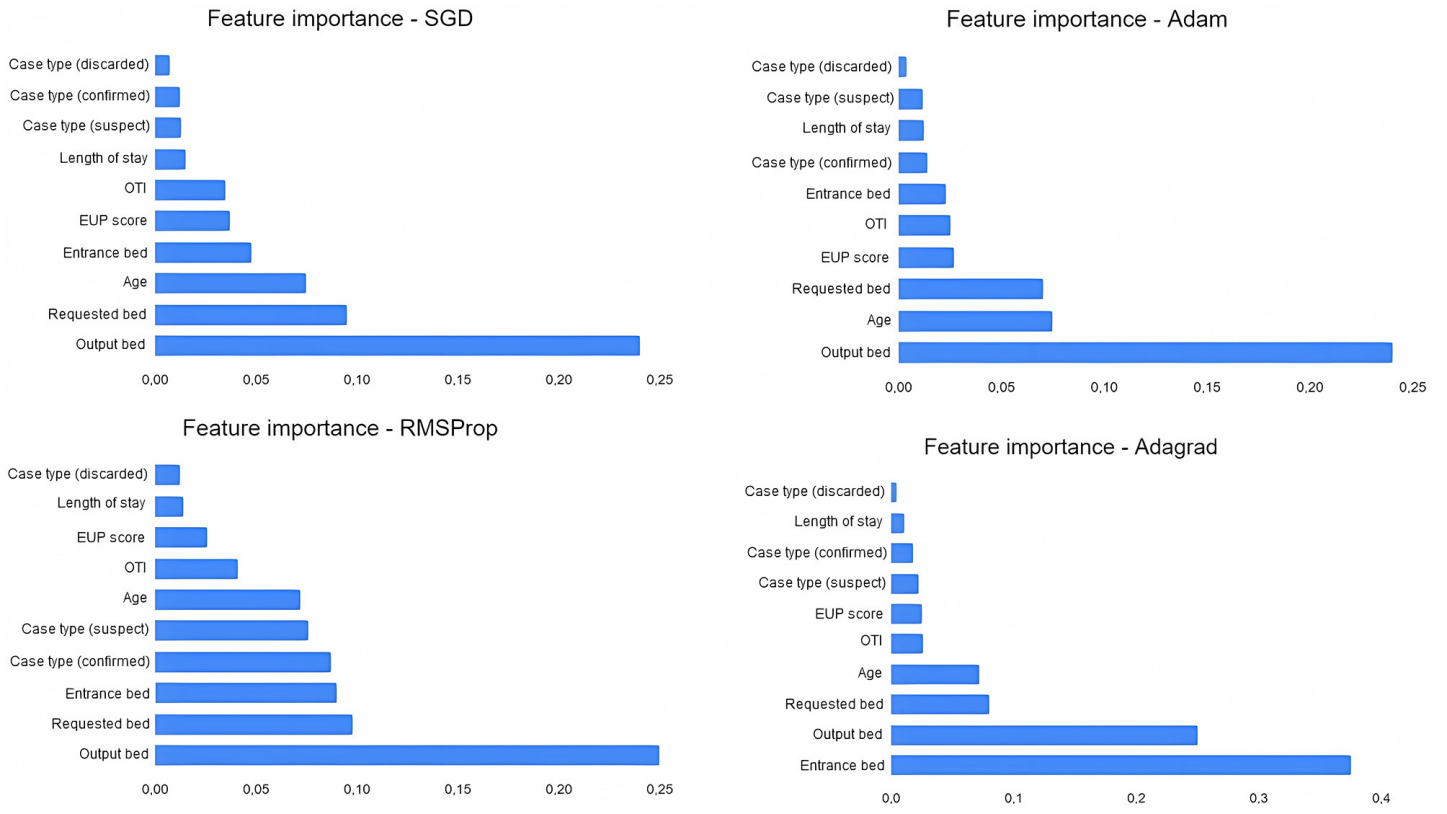
Features importances of multiperceptron layers models (deep learning). The figure shows the SGD optimizer, followed by Adam, RMSProp, and Adagrad.

When evaluating the *features importances* of the tree models (Figure 5), there is a convergence of results. The characteristics of *output bed, age*, and *length of stay* are among the most important for model selection. The *EUP score* is also relevant between the models, although the *entrance bed* and *requested bed* have a greater influence on Random Forest. The *type of case* and whether the patient was under orotracheal intubation during the request were not very decisive for the models. In the MLP models (Figure 6), the types *requested bed entrance bed* and *output bed* are among the most relevant characteristics for RMSProp and Adagrad, while SGD and Adam consider *output bed, requested bed*, and *age* to be the most relevant ones. Among all the models, the type of case (discarded) is the least important variable in determining the outcome. It was noted that length of stay stands out more in Decision Tree and Random Forest decision-making, while in the other classifiers, it is a variable that is not highly valued.

Comparing our results with the correlation of variables from the Phik model, the deep learning models selected the most important features similarly to the correlations presented; *output bed, requested bed*, and *entrance bed* are among the most relevant in RMSProp and Adagrad; *output bed* and *requested bed* appear among the most relevant for SGD and Adam. This correspondence points to a relational convergence between Phik and the best selection of variables to determine the outcome carried out by MLP models.

**Figure 7:**
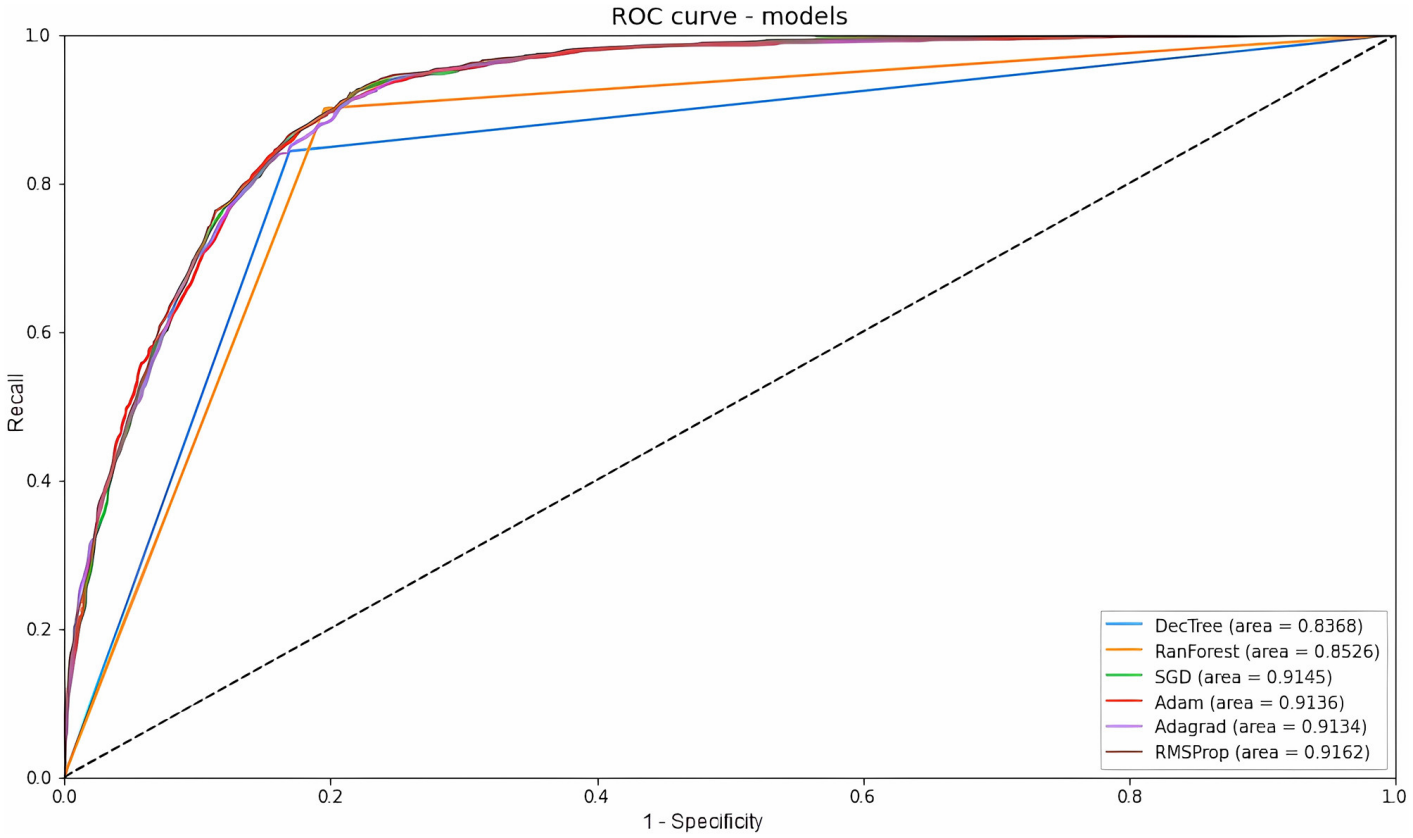
ROC curve and AUC value of all models.

ROC and AUC are efficient techniques for summarizing the prediction accuracy of models. The ROC is obtained from the ratio between the recall and the complement of specificity, while the area under the curve varies from 0 to 1, indicating a totally correct prediction or not (Shanbehzadeh et al., 2022). The machine learning ROC-AUC models obtained similar results to each other. Random Forest showed the best classification of true and false positives with an AUC of 0.852, Decision Tree showed an AUC of 0.843, a result that was expected given that it had the lowest average recall. Meanwhile, among the deep learning classifiers, Adagrad had the lowest value (AUC = 0.912), followed by SGD (AUC = 0.913), Adam (AUC = 0.914), and RMSProp (0.916). All these data are displayed in Figure 7. Overall, an AUC of 0.7 to 0.8 is considered acceptable, 0.8 to 0.9 is considered excellent, and above 0.9 is considered outstanding (Shanbehzadeh et al., 2022).

### 3.3 Analysis of results

Investments in efficient health systems have been decisive for the better performance of several regions in acquiring data for decision-making in the midst of the pandemic. In addition, the use of artificial intelligence systems has been widely disseminated in academic literature as an aid in various issues to combat COVID-19, whether through patient health data (Debnath et al., 2020; Moulaei et al., 2022; Shanbehzadeh et al., 2022), interventions with radiological images (Ahsan et al., 2020; Ghaderzadeh et al., 2021a, 2022), dissemination of false information about COVID-19 (Endo et al., 2022), forecast of the number of ICU beds in times of crisis (Goic et al., 2021), predicting the time needed to transfer a patient to an ICU bed (Cheng et al., 2020), among others.

Our results compared the performance of six different computational methods in predicting patient mortality from bed regulation data. As for the machine learning models, Random Forest showed the best results in terms of accuracy (82.97%), precision (79.35%), recall (84.80%), F1-score (80.74%), Specificity (84.79%), and ROC-AUC (85.2%). The good performance of this classifier is also highlighted in the work of Prakash et al. (2020), Gupta et al. (2021), Endo et al. (2022), and several others in the literature. As for the deep learning models, SGD showed the best results in terms of accuracy (84.01%), precision (79.57%) and F1-score (81.00%), and RMSprop showed the highest recall (84.67%), specificity (84.67%), and ROC-AUC (91.6%). The selection of SGD as the top optimizer for increasing accuracy also appears in the work of Andrade et al. (2022), however, it does not converge with the work of Ahsan et al. (2020), in which SGD was the worst optimizer selected. Nevertheless, it should be noted that the variability of the data model used to structure the neural network can produce different results, so experimentation with different classifiers is essential to determine the best one.

The MLP with SGD optimizer proved to be the best model for determining the outcome of discharge and death and in terms of assertiveness for predicting discharges. Therefore, if the health professional's goal is to determine whether that patient will have a positive outcome, the SGD is the most recommended optimizer. Whereas, if the operator's objective is to identify which of the total samples selected were best classified, RMSProp is the most recommended optimizer.

When it comes to decision-making, besides the model assertively predicting the outcome, it is also interesting to minimize the worst-case scenarios. In a pandemic scenario, where there is great competition for the number of beds, it is important that the model minimizes situations in which there would be a death and the model classified it as a discharge (false negatives) because this would ensure better management and efficiency of bed allocation so that a patient who had a greater chance of a positive outcome was referred. For this reason, besides having high accuracy and precision, recall and ROC-AUC should be maximized. Similarly, a patient who would have been discharged and the tool classified as death could lead the regulator to make the wrong decision, sending a patient who had a chance of survival to palliative care. Thus, we recommend a joint evaluation of the results of the SGD and RMSProp for a definitive decision.

It is important to highlight that all the data used are real-world data and the variables length of stay and output bed are not controlled data. Therefore, we carried out new tests to ensure that there would be no variability in our metrics, zeroing the length of stay and keeping the output bed the same as the entrance bed. And as expected, there were no significant changes in the metrics of the models evaluated. This is because these models aim, based on the patient's clinical data, to evaluate whether the choice of bed had a better outcome and, consequently, a better length of stay. In other words, dwell time, and outcome are expected results of our model. So, what actually happens is that the model looks at the patient's clinical parameters to analyze whether the choice of bed was the most appropriate for a better outcome and shorter length of stay.

## 4 Discussion

The proposed models enhance the decision-making process of the regulatory professional, to reduce the subjectivity of indicating a patient who would have a better chance of survival for a given hospital bed. Muhammad et al. (2021) also point to a reduction in the burden on care operators and promote more effective care. It must be emphasized that the medical appraisal should be sovereign in all situations so that the models can only suggest the best course of action. Moreover, models have the added benefit of being flexible to changes, allowing for different forecasts (Subudhi et al., 2020). That is, if during the regulation process, there is a need to adjust some new information about the regulation process, the model is able to suggest, in real time, a new forecast for that case.

In the context of global health to tackle COVID-19, widespread investment in health sectors is essential. Brazil already experienced bed overcrowding even before the health crisis (Soares, 2017), thus a difficult performance in dealing with the pandemic was expected. When comparing the situation in Europe, which began a policy of reducing the number of active beds between 2010 and 2017 due to the low use of hospitalization services, Pecoraro, Luzi, and Clemente (Pecoraro et al., 2021) showed an explicit relationship between the increase in investment in the health area and its results in the face of health crises. It was found that Germany invested more in health policies, the number of professionals, and the number of beds, and achieved a more significant performance in minimizing the effects of the pandemic compared to Spain, France, and Italy.

With regard to digital health, there has been pressure for government entities to adopt efficient strategies by implementing computerized systems that help obtain relevant information for the population and assist decision-makers and health agents (Budd et al., 2020; Valentim et al., 2021). For this to be possible, maintaining transparency as well as a cyclical and incremental evolution is fundamental to guaranteeing quality data, with the aim of understanding the scenario in due course (Rasheed et al., 2020; Valentim et al., 2021). The RegulaRN and other systems are examples of this, so utilizing its data to strengthen future decision-making in the bed regulation process corroborates the expectations proposed by the academic, governmental, and social community.

This paper presents the results of an epidemiological analysis of bed regulation data combined with a methodology of computational models. Thus, aside from presenting new information based on COVID-19, the main characteristics of the inpatient outcome were identified. The data allowed us to present mean and absolute values with statistical significance superior to other studies (Baqui et al., 2021), besides making it possible to suggest a risk criterion scale, including the independent variables age and length of stay, which can be used by health units during the hospitalization process. This scale may contribute to the process of providing beds so that patients with the highest values on the scale would be allocated to palliative care beds.

This work complies with the first three stages (data preparation, model development, and model validation) suggested by the review work proposed by de Hond et al. (2022) and is under development to be integrated into RegulaRN, to ensure compliance with the stages of software, evaluation, and implementation in daily healthcare practice.

Furthermore, the use of computational methods can be of great value in the regulatory management of patients with COVID-19. The selected models showed good values for the main metrics evaluated and could optimize the work of regulators and minimize failures in terms of the real outcome of the patient or the government's costs in funding hospital articles and beds in an expensive manner. It should be emphasized that the costs involved in keeping a patient in an intensive care unit bed are substantially higher than in a ward. Moreover, the results indicated by the models themselves may indirectly indicate the need for government action to open or close new beds.

This model, presented in this article, was validated together with the technical team of the Public Health Secretariat of the State of Rio Grande do Norte. Therefore, it is incorporated into RegulaRN with the aim of helping regulatory doctors make decisions. According to the selected characteristics, the models can be consulted by regulatory doctors at any stage of the regulation, whether at the first indication (when the patient has not yet been assigned to a bed), or also when the patient is already in a bed (already hospitalized) to use the data generated to suggest a new outcome prediction. It is noteworthy that in addition to the team of regulatory doctors, this is an important tool for health managers, as they now obtain timely information (on-time) for decision-making and the formulation of public health policies that can guarantee better access to health services.

Among the limitations of our study and the possibility of future work, is to integrate and interoperate with the patients' vaccination data, because Brazil started the vaccination process against COVID-19 in January 2021. In this sense, even those who may have been partially immunized and needed hospitalization may contain enough antibodies to promote a different outcome than a patient without any dose of vaccine (Sales-Moioli et al., 2022). Furthermore, the database sent by the health department, which was used in this research, did not have a breakdown of patients by gender, so this analysis was not included in this first work but has already been requested for future work. Finally, another limitation of the article is to better work with censored data on output bed and length of stay, considering that these are variables that occur stochastically. Therefore, during the initial regulation process, the output bed and length of stay are information that is not yet completely defined, requiring some time for the model to be able to present a good prediction quality.

## 5 Conclusions

This study used the RegulaRN database from April 2020 to August 2022 to select computational models based on artificial intelligence to predict the outcome of patients who were regulated during the COVID-19 pandemic in the state of Rio Grande do Norte. Among the selected models, the MLP with SGD optimizer obtained the highest accuracy, precision, and f1 score for predicting the outcome (discharge or death), selecting the output bed, requested bed, and age as the most relevant. On the other hand, RMSprop obtained higher scores in recall, specificity, and ROC-AUC, selecting output bed, requested bed, and entry bed as important variables for the outcome. In addition, we propose a scale of risk criteria that can be used by healthcare facilities to control and make beds available.

The models can be used by the regulation center to assist regulatory professionals during the indication for a bed, increasing the assertiveness of the patient's referral and minimizing the impacts of mistaken regulation, i.e., directing a patient with a lower chance of survival to an ICU bed when the best option would be palliative care, or referring a patient who can be treated in a ward bed to an ICU. In a pandemic situation, in addition to the effects on human health, the computer model could help government decisions on the costs involved with hospital beds.

## Data availability statement

The datasets presented in this study can be found in online repositories. The names of the repository/repositories and accession number(s) can be found below: https://zenodo.org/record/8122564.

## Ethics statement

The requirement of ethical approval was waived by Research Ethics Committee (CEP) of the Federal University of Rio Grande do Norte for the studies involving humans because according to resolution 674, 2022 of the National Health Council (NHC) of the Ministry of Health (MoH) this research is exempt from registration with the Research Ethics Committee (CEP)/Brazil or the National Research Ethics Commission (CONEP)/Brazil. The studies were conducted in accordance with the local legislation and institutional requirements. Written informed consent for participation in this study was provided by the participants' legal guardians/next of kin.

## Author contributions

TB: Conceptualization, Data curation, Formal analysis, Methodology, Software, Writing—original draft, Writing—review & editing. NV: Conceptualization, Data curation, Methodology, Writing—original draft, Writing—review & editing. PC: Conceptualization, Data curation, Methodology, Writing—review & editing. FF: Data curation, Methodology, Writing—review & editing. LM: Data curation, Methodology, Software, Writing—review & editing. MB: Conceptualization, Methodology, Writing—review & editing. FA: Conceptualization, Methodology, Writing—review & editing. CP: Investigation, Methodology, Writing—review & editing. IS-G: Data curation, Methodology, Software, Writing—review & editing. GS: Data curation, Methodology, Software, Writing—review & editing. LR: Data curation, Methodology, Software, Writing—review & editing. AM: Methodology, Supervision, Writing—review & editing. JS: Conceptualization, Supervision, Writing—review & editing. JP: Conceptualization, Supervision, Writing—review & editing. IA: Writing—review & editing, Formal analysis, Supervision. RV: Funding acquisition, Methodology, Project administration, Supervision, Writing—original draft, Writing—review & editing.

## References

[B1] Ahsan M. M. E Alam T. Trafalis T. Huebner P. (2020). Deep mlp-cnn model using mixed-data to distinguish between COVID-19 and non-COVID-19 patients. Symmetry 12, 1526. 10.3390/sym12091526

[B2] Andrade E. C. d. Pinheiro P. R. Barros A. L. B. d. P. Nunes L. C. Pinheiro L. I. C. Pinheiro P. G. C. D.. (2022). Towards machine learning algorithms in predicting the clinical evolution of patients diagnosed with covid-19. Appl. Sci. 12, 8939. 10.3390/app12188939

[B3] Aquino E. M. Silveira I. H. Pescarini J. M. Aquino R. Souza-Filho J. A. Rocha A. S.. (2020). Social distancing measures to control the COVID-19 pandemic: potential impacts and challenges in brazil. Ciencia Saude Colet. 25, 2423–2446. 10.1590/1413-81232020256.1.10502020 32520287

[B4] Baqui P. Marra V. Alaa A. M. Bica I. Ercole A. van der Schaar M. (2021). Comparing COVID-19 risk factors in brazil using machine learning: the importance of socioeconomic, demographic and structural factors. Scient. Rep. 11, 15591. 10.1038/s41598-021-95004-8 34341397 PMC8329284

[B5] Bastos S. B. Cajueiro D. O. (2020). Modeling and forecasting the early evolution of the COVID-19 pandemic in brazil. Scient. Rep. 10, 19457. 10.1038/s41598-020-76257-1 33173127 PMC7655855

[B6] Bergstra J. Bengio Y. (2012). Random search for hyper-parameter optimization. J. Mach. Lear. Res. 13, 281–305.

[B7] Bian J. Modave F. (2020). The rapid growth of intelligent systems in health and health care. Health Inform. J. 26, 5–7. 10.1177/1460458219896899 31928307

[B8] Budd J. Miller B. S. Manning E. M. Lampos V. Zhuang M. Edelstein M.. (2020). Digital technologies in the public-health response to COVID-19. Nat. Med. 26, 1183–1192. 10.1038/s41591-020-1011-4 32770165

[B9] Charbuty B. Abdulazeez A. (2021). Classification based on decision tree algorithm for machine learning. J. Appl. Sci. Technol. Trends 2, 20–28. 10.38094/jastt20165

[B10] Chawla N. V. Bowyer K. W. Hall L. O. Kegelmeyer W. P. (2002). Smote: synthetic minority over-sampling technique. J. Artif. Intell. Res. 16, 321–357. 10.1613/jair.953

[B11] Cheng F.-Y. Joshi H. Tandon P. Freeman R. Reich D. L. Mazumdar M.. (2020). Using machine learning to predict icu transfer in hospitalized COVID-19 patients. J. Clin. Med. 9, 1668. 10.3390/jcm9061668 32492874 PMC7356638

[B12] Costa K. T. S. Morais T. N. B. d. Justino D. C. P. Andrade F. B. (2021). Evaluation of the epidemiological behavior of mortality due to COVID-19 in Brazil: a time series study. PLoS ONE 16, e0256169. 10.1371/journal.pone.0256169 34383857 PMC8360598

[B13] Cotrim Junior D. F. Cabral L. M. d. S. (2020). Crescimento dos leitos de uti no país durante a pandemia de COVID-19: desigualdades entre o público x privado e iniquidades regionais. Phys. Rev. Saúde Colet. 30, e300317. 10.1590/s0103-73312020300317

[B14] Dashboard W. C. C. (2022). Who coronavirus (COVID-19) dashboard. Available online at: https://covid19.who.int/ (accessed October 20, 2022).

[B15] Dauphin Y. De Vries H. Bengio Y. (2015). “Equilibrated adaptive learning rates for non-convex optimization,” in Advances in Neural Information Processing Systems 28.

[B16] de Hond A. A. Leeuwenberg A. M. Hooft L. Kant I. M. Nijman S. W. van Os H. J.. (2022). Guidelines and quality criteria for artificial intelligence-based prediction models in healthcare: a scoping review. NPJ Digital Med. 5, 2. 10.1038/s41746-021-00549-7 PMC8748878 35013569

[B17] Debnath S. Barnaby D. P. Coppa K. Makhnevich A. Kim E. J. Chatterjee S.. (2020). Machine learning to assist clinical decision-making during the COVID-19 pandemic. Bioelectr. Med. 6, 1–8. 10.1186/s42234-020-00050-8 32665967 PMC7347420

[B18] Duchi J. Hazan E. Singer Y. (2011). Adaptive subgradient methods for online learning and stochastic optimization. J. Mach. Learn. Res. 12, 2121–2159.

[B19] Endo P. T. Santos G. L. de Lima Xavier M. E. Nascimento Campos G. R. de Lima L. C. Silva I.. (2022). Illusion of truth: analysing and classifying COVID-19 fake news in brazilian portuguese language. Big Data Cogn. Comput. 6, 36. 10.3390/bdcc6020036

[B20] Ensor K. B. Glynn P. W. (1997). Stochastic optimization via grid search. Appl. Mathem. Am. Mathem. Soc. 33, 89–100.

[B21] Fernández A. Garcia S. Herrera F. Chawla N. V. (2018). Smote for learning from imbalanced data: progress and challenges, marking the 15-year anniversary. J. Artif. Intell. Res. 61, 863–905. 10.1613/jair.1.11192

[B22] Ghaderzadeh M. Aria M. (2021). “Management of COVID-19 detection using artificial intelligence in 2020 pandemic,” in Proceedings of the 5th International Conference on Medical and Health Informatics 32–38. 10.1145/3472813.3472820

[B23] Ghaderzadeh M. Aria M. Asadi F. (2021a). X-ray equipped with artificial intelligence: changing the COVID-19 diagnostic paradigm during the pandemic. BioMed. Res. Int. 2021, 9942873. 10.1155/2021/9942873 34458373 PMC8390162

[B24] Ghaderzadeh M. Asadi F. Jafari R. Bashash D. Abolghasemi H. Aria M. (2021b). Deep convolutional neural network-based computer-aided detection system for COVID-19 using multiple lung scans: design and implementation study. J. Med. Internet Res. 23, e27468. 10.2196/27468 33848973 PMC8078376

[B25] Ghaderzadeh M. Eshraghi M. A. Asadi F. Hosseini A. Jafari R. Bashash D.. (2022). Efficient framework for detection of COVID-19 omicron and delta variants based on two intelligent phases of cnn models. Comput. Mathem. Methods Med. 2022, 4838009. 10.1155/2022/4838009 35495884 PMC9050257

[B26] Goic M. Bozanic-Leal M. S. Badal M. Basso L. J. (2021). Covid-19: Short-term forecast of icu beds in times of crisis. PLoS ONE 16, e0245272. 10.1371/journal.pone.0245272 33439917 PMC7806165

[B27] Grandini M. Bagli E. Visani G. (2020). Metrics for multi-class classification: an overview. arXiv preprint arXiv:2008.05756.

[B28] Gupta V. K. Gupta A. Kumar D. Sardana A. (2021). Prediction of COVID-19 confirmed, death, and cured cases in india using random forest model. Big Data Min. Analy. 4, 116–123. 10.26599/BDMA.2020.9020016

[B29] Hu B. Guo H. Zhou P. Shi Z.-L. (2021). Characteristics of SARS-CoV-2 and COVID-19. Nat. Rev. Microbiol. 19, 141–154. 10.1038/s41579-020-00459-7 33024307 PMC7537588

[B30] Huang C. Wang Y. Li X. Ren L. Zhao J. Hu Y.. (2020). Clinical features of patients infected with 2019 novel coronavirus in Wuhan, China. Lancet 395, 497–506. 10.1016/S0140-6736(20)30183-5 31986264 PMC7159299

[B31] Jia W. Sun M. Lian J. Hou S. (2022). Feature dimensionality reduction: a review. Complex Intell. Syst. 8, 2663–2693. 10.1007/s40747-021-00637-x

[B32] Kingma D. P. Ba J. (2014). Adam: Amazon.com method for stochastic optimization. arXiv preprint arXiv:1412.6980.

[B33] Lino D. O. d. C Barreto R. Souza F. D. d. Lima C. J. M. d.. (2020). Impact of lockdown on bed occupancy rate in a referral hospital during the COVID-19 pandemic in northeast Brazil. Brazil. J. Infect. Dis. 24, 466–469. 10.1016/j.bjid.2020.08.002 32888904 PMC7457936

[B34] Lipton Z. C. Elkan C. Naryanaswamy B. (2014). “Optimal thresholding of classifiers to maximize F1 measure”, in Machine Learning and Knowledge Discovery in Databases: European Conference, ECML PKDD 2014, (Nancy: Springer), 225–239. 10.1007/978-3-662-44851-9_15 PMC4442797 26023687

[B35] Maldonado R. N. Savio R. O. Feijó V. B. E. R. Aroni P. Rossaneis M. A. Haddad M.. (2021). Hospital indicators after implementation of bed regulation strategies: an integrative review. Rev. Brasil. de Enferm. 74, e20200022. 10.1590/0034-7167-2020-0022 34161538

[B36] Moulaei K. Shanbehzadeh M. Mohammadi-Taghiabad Z. Kazemi-Arpanahi H. (2022). Comparing machine learning algorithms for predicting COVID-19 mortality. BMC Med. Inform. Decis. Making 22, 1–12. 10.1186/s12911-021-01742-0 34983496 PMC8724649

[B37] Muhammad L. Algehyne E. A. Usman S. S. Ahmad A. Chakraborty C. Mohammed I. A. (2021). Supervised machine learning models for prediction of COVID-19 infection using epidemiology dataset. SN Comput. Sci. 2, 1–13. 10.1007/s42979-020-00394-7 33263111 PMC7694891

[B38] Pecoraro F. Luzi D. Clemente F. (2021). The efficiency in the ordinary hospital bed management: a comparative analysis in four european countries before the COVID-19 outbreak. PLoS ONE 16, e0248867. 10.1371/journal.pone.0248867 33750956 PMC7984624

[B39] Pedregosa F. Varoquaux G. Gramfort A. Michel V. Thirion B. Grisel O.. (2011). Scikit-learn: machine learning in python. J. Mach. Learn. Res. 12, 2825–2830.

[B40] Perondi B. Miethke-Morais A. Montal A. C. Harima L. Segurado A. C. (2021). Setting up hospital care provision to patients with COVID-19: lessons learnt at a 2400-bed academic tertiary center in Sao Paulo, Brazil. Brazil. J. Infect. Dis. 24, 570–574. 10.1016/j.bjid.2020.09.005 33157034 PMC7604059

[B41] Prakash K. B. Imambi S. S. Ismail M. Kumar T. P. Pawan Y. (2020). Analysis, prediction and evaluation of COVID-19 datasets using machine learning algorithms. Int. J. 8, 2199–2204. 10.30534/ijeter/2020/117852020

[B42] Rasheed J. Jamil A. Hameed A. A. Aftab U. Aftab J. Shah S. A.. (2020). A survey on artificial intelligence approaches in supporting frontline workers and decision makers for the COVID-19 pandemic. Chaos, Solit. Fractals 141, 110337. 10.1016/j.chaos.2020.110337 33071481 PMC7547637

[B43] Rees E. M. Nightingale E. S. Jafari Y. Waterlow N. R. Clifford S. B.. (2020). COVID-19 length of hospital stay: a systematic review and data synthesis. BMC Med. 18, 1–22. 10.1186/s12916-020-01726-3 32878619 PMC7467845

[B44] Sales-Moioli A. I. L. Galv ao-Lima L. J. Pinto T. K. Cardoso P. H. Silva R. D. Fernandes F.. (2022). Effectiveness of COVID-19 vaccination on reduction of hospitalizations and deaths in elderly patients in Rio Grande do Norte, Brazil. Int. J. Environ. Res. Public Health 19, 13902. 10.3390/ijerph192113902 36360782 PMC9653712

[B45] Shahzad A. Zafar B. Ali N. Jamil U. Alghadhban A. J. Assam M.. (2022). COVID-19 vaccines related users response categorization using machine learning techniques. Computation 10, 141. 10.3390/computation10080141

[B46] Shailaja K. Seetharamulu B. Jabbar M. (2018). “Machine learning in healthcare: a review,” in 2018 Second International Conference on Electronics, Communication and Aerospace Technology (ICECA) (IEEE), 910–914. 10.1109/ICECA.2018.8474918

[B47] Shanbehzadeh M. Yazdani A. Shafiee M. Kazemi-Arpanahi H. (2022). Predictive modeling for COVID-19 readmission risk using machine learning algorithms. BMC Med. Inform. Decis. Mak. 22, 139. 10.1186/s12911-022-01880-z 35596167 PMC9122247

[B48] Soares V. S. (2017). Analysis of the internal bed regulation committees from hospitals of a southern brazilian city. Einstein 15, 339–343. 10.1590/s1679-45082017gs3878 29091157 PMC5823049

[B49] Sokolova M. Lapalme G. (2009). A systematic analysis of performance measures for classification tasks. Inform. Process. Manage. 45, 427–437. 10.1016/j.ipm.2009.03.002

[B50] Subudhi S. Verma A. Patel A. B. (2020). Prognostic machine learning models for COVID-19 to facilitate decision making. Int. J. Clin. Pract. 74, e13685. 10.1111/ijcp.13685 32810316 PMC7461008

[B51] Sutskever I. Martens J. Dahl G. Hinton G. (2013). “On the importance of initialization and momentum in deep learning,” in International Conference on Machine Learning (PMLR), 1139–1147.

[B52] Valentim R. A. d. M Lima T. S. Cortez L. R. Barros D. M. d. S. Silva R. D. d.. (2021). The relevance a technology ecosystem in the brazilian national health services COVID-19 response: the case of Rio Grande do Norte, Brazil. Ciênc. Saúde Colet. 26, 2035–2052. 10.1590/1413-81232021266.44122020 34231717

[B53] Vaughan L. Zhang M. Gu H. Rose J. B. Naughton C. C. Medema G.. (2023). An exploration of challenges associated with machine learning for time series forecasting of COVID-19 community spread using wastewater-based epidemiological data. Sci. Total Environ. 858, 159748. 10.1016/j.scitotenv.2022.159748 36306840 PMC9597519

[B54] Vekaria B. Overton C. Wiśniowski A. Ahmad S. Aparicio-Castro A. Curran-Sebastian J.. (2021). Hospital length of stay for COVID-19 patients: data-driven methods for forward planning. BMC Infect. Dis. 21, 1–15. 10.1186/s12879-021-06371-6 34294037 PMC8295642

[B55] Yu L. Halalau A. Dalal B. Abbas A. E. Ivascu F. Amin M.. (2021). Machine learning methods to predict mechanical ventilation and mortality in patients with COVID-19. PLoS ONE 16, e0249285. 10.1371/journal.pone.0249285 33793600 PMC8016242

[B56] Zhang C. Liao Q. Rakhlin A. Miranda B. Golowich N. Poggio T. (2018). Theory of deep learning iib: Optimization properties of sgd. arXiv preprint arXiv:1801.02254.

[B57] Zhu N. Zhang D. Wang W. Li X. Yang B. Song J.. (2020). A novel coronavirus from patients with pneumonia in China, 2019. New Engl. J. Med. 382, 727–733. 10.1056/NEJMoa2001017 31978945 PMC7092803

